# Preparing medical students for their educational task as physicians: important, desirable and unexplored territory

**DOI:** 10.1186/s12909-024-05328-y

**Published:** 2024-04-09

**Authors:** Bas PH ter Brugge, Lena Sophia Fegg, Marjo Wijnen-Meijer

**Affiliations:** 1 Vilente, Doorwerth, Netherlands; 2 School of Medicine and Health, Technical University of Munich, TUM Medical Education Center, Munich, Germany; 3 Institute of Medical Education, Medical Faculty and University Hospital Carl Gustav Carus, TUD Dresden University of Technology, Fetscherstraße 74, 01307 Dresden, Germany

**Keywords:** Peer-teaching, Medical students, Education, Teaching, Learning, Educational skills, Competencies, Physicians

## Abstract

**Background:**

Physicians engage in educational activities in daily practice and take over an important role in providing information and transferring knowledge to patients and medical students. Therefore, it is important to focus on methods to develop teaching skills during medical school. Peer-teaching is a teaching method that is connected to different positive learning outcomes. This study aims to investigate the perspective of medical students regarding teaching as a core competency of physicians and peer-teaching as an opportunity to acquire educational skills. The study also aims to examine to what extent medical students are prepared for their teaching role at medical schools.

**Methods:**

This cross-sectional study was performed by an online survey amongst Dutch medical students from all medical schools across all years of study. In total, 2666 medical students filled out the survey. The survey was part of the annual online survey of the Dutch medical advocacy group (DeGeneeskundestudent) amongst all medical students in the fall of 2017. The data were analysed with descriptive statistics and statistical tests (chi-squared-test and binomial test).

**Results:**

The results show that 49% of medical students see teaching as one of the core tasks of a physician. However, only 25% feel well prepared by their medical school for this teaching role. Instead, there are many students who gain experiences and teaching skills on their own outside medical schools. 64% of the respondents agrees that senior medical students can educate junior medical students well.

**Conclusions:**

Implementing peer-teaching in the curricular of medical schools could be an effective teaching method to prepare medical students for their future teaching role. It is important that medical schools focus on enhancing educational quality and designing learning environments for best learning outcomes to better prepare medical students for professional life.

## Background

The teaching role of physicians is a core competency in the new Dutch Medical Training Framework: “Physicians contribute as academics to the application, spread, translation and proliferation of knowledge in practice through lifelong learning, training others, evaluating evidence and contributing to scientific research” [1]. Every physician must be able to “create a safe learning environment”, “provide a teaching activity” and “constructively evaluate teaching activities to improve education” [1]. After all, every physician engages in educational activities in one way or another. It has been shown that a general practitioner spends up to 20% of his consultation time on patient education and a medical specialist up to 10% of his time on supervising residents or medical students [2, 3]. Physicians play an important role in providing information and transferring knowledge to patients and medical students. Therefore, parallel to clinical skills the acquisition of educational skills should begin in medical school and continue throughout postgraduate training [4].

Educational skills are best developed by doing it yourself [5, 6]. Peer-teaching, i.e. students teaching other students, is a method for medical students to practice teaching in a controlled environment [6]. In some medical faculties, both in the Netherlands and other countries, peer-teaching is a regular part of medical school [7, 8]. To develop medical students’ teaching skills, peer-teaching programmes, teaching workshops, and community outreach programmes are used [9]. Many medical schools in the United States offer formal students-as-teachers (SAT) programmes, where students are assigned educational roles such as peer mentors, teaching assistants or contributing to the development of a curriculum design. These programmes benefit the students’ teaching skills, improve their clinical knowledge and communication- and professional skills. Peer-teachers can benefit from peer-teaching experiences in many ways. Teaching offers a chance to identify personal strengths and weaknesses by preparing complex medical knowledge, organizing classes, enhancing public speaking skills, giving- and receiving feedback, working in a team and leading near-peer students [6–8, 10, 11]. By actively participating in their training the medical students’ intrinsic motivation is improved [12, 13].

In a recent non-randomized controlled trial by Veloso et al. (2019), it was shown that medical students who taught Basic Life Support skills to community health professionals had a better theoretical and practical performance in Basic Life Support, than medical students who didn’t teach these skills [14]. Peer-teaching is further supported by studies that have found no difference in medical students’ academic achievements when taught by peer-teachers or faculty staff. While peer-teachers are considered less knowledgeable than faculty staff, students actually feel more at ease asking questions and, due to peer-teachers being regarded as more approachable, they are better understood and guided in comprehending difficult topics [11, 14–16]. A final reason for implementation of peer -teaching programmes is the rise in student numbers. Peer-teachers offer a solution to overcome the strained teaching capacity of faculty staff [11, 17].

There is evidence that former peer-teaching physicians become more engaged in educational activities. A study by Kloek et al. (2016) indicated that these physicians themselves highly appreciated the teaching internship and are likely committed to building an educational career in their future professional life [18].

Unfortunately, little is known about the perspective of medical students regarding teaching as a physician and peer-teaching. This perspective is relevant to facilitate the introduction of peer-teaching by medical schools and better prepare medical students for their future teaching role as a physician. It is relevant to assess medical students’ perspective on the teaching role of physicians and their educational activities during medical school. Therefore, this study aims to gain insight into medical students´ opinion on teaching as a physician and peer teaching by answering the following research questions:

- To what extent do medical students consider teaching a core competency of a physician?
- How and to what extent are medical students prepared for teaching as a physician during medical school?

## Methods

### Study design and participants

This study has a cross-sectional design and is performed by an online survey amongst medical students.

### Population

The research population comprised of Dutch medical students from all medical schools across all years of study. In the Netherlands, there are eight medical schools that offer a six-year undergraduate medical training. The undergraduate program is divided in a three year Bachelor, with mostly theoretical education, and a three year Master, with both theoretical educations and clerkships.

### Survey

The survey started with a general section on gender, university and study-phase. Next, five questions asked for the participants view regarding (the preparation for) teaching as a physician and peer-teaching (see Tables 1 and 2). The questions were grounded in literature [17]. Four questions were answered on a five-point Likert scale (strongly agree- strongly disagree), in which answer option 3 means “neutral” and for the question “older students can teach younger year medical students well” also “no experience”. The final question was a binary question (yes/no).

**Table 1:** Answers to the questions, by phase of study and gender

Result*	Number of students; % (*n*/*N*)^‡^				
	Teaching is part of the core duties of a physician				
	Total (*n* = 2493)	Men (*n* = 553)	Women (*n* = 1940)	Bachelor (*n* = 1269)	Master (*n* = 1224)
Agree	49.4 (1232)	**57.7 (319)** ^**†**^	47.1 (913)	35.1 (446)	**64.2 (786)** ^**†**^
Neutral	28.8 (719)	24.1 (133)	**30.2 (586)** ^**†**^	**33.6 (427)** ^**†**^	23.9 (292)
Disagree	21.7 (542)	18.3 (101)	**22.7 (441)** ^**†**^	**31.2 (396)** ^**†**^	11.9 (146)
	**Older year medical students can teach younger year medical students well**				
	Total (*n* = 2490)	Men (*n* = 552)	Women (*n* = 1938)	Bachelor (*n* = 1267)	Master (*n* = 1223)
Agree	63.6 (1584)	66.7 (368)	62.7 (1216)	62 (786)	65.2 (798)
Neutral	24.3 (604)	21.9 (121)	24.9 (483)	**26.6 (337)** ^**†**^	21.9 (267)
Disagree	12.1 (302)	11.4 (63)	12.3 (239)	11.4 (134)	**12.9 (158)** ^**†**^
	**I feel well prepared to teach as a physician from the medical school**				
	Total (*n* = 2486)	Men (*n* = 552)	Women (*n* = 1934)	Bachelor (*n* = 1263)	Master (*n* = 1223)
Agree	26.5 (658)	**36.2 (200)** ^**†**^	23.7 (458)	26.7 (337)	26.2 (321)
Neutral	34.5 (858)	29.9 (165)	**35.8 (693)** ^**†**^	**40.9 (516)** ^**†**^	28 (342)
Disagree	39 (970)	33.9 (187)	**40.5 (783)** ^**†**^	32.5 (410)	**45.8 (560)** ^**†**^
	**I feel well prepared to teach as a physician based on my own experience**				
	Total (*n* = 2479)	Men (*n* = 550)	Women (*n* = 1929)	Bachelor (*n* = 1258)	Master (*n* = 1221)
Agree	47.6 (1180)	**61.5 (338)** ^**†**^	43.6 (842)	39.7 (499)	**55.8 (681)** ^**†**^
Neutral	30.4 (758)	26 (143)	**31.7 (611)** ^**†**^	**35.7 (449)** ^**†**^	25 (305)
Disagree	22 (545)	12.5 (69)	**24.7 (476)** ^**†**^	**24.6 (310)** ^**†**^	19.2 (235)
Missing; n (%)	173–187 (6.5–7)	**53–56 (8.7–9.2)** ^**†**^	120–131 (5.8–6.4)	**140–151 (9.9–10.7)** ^**†**^	33–36 (2.6–2.9)

* Disagree = totally disagree (response option 1) / disagree (response option 2)

Neutral = disagree/disagree (answer option 3). For question ‘’older year medical students can teach younger year medical students well’’ including ´no experience’.

Agree = agree (response option 4) / totally agree (response option 5)

† Significant for *p* < 0.05 (rows)

**Table 2:** Answers to the questions, by university

Result*	Number of students; % (n/N)^‡^									
	Teaching is part of the core duties of a physician									
	Total (*n* = 2493)	UvA (*n* = 238)	VU (*n* = 294)	Groningen (*n* = 425)	Leiden (*n* = 288)	Maastricht (*n* = 310)	Nijmegen (*n* = 358)	Rotterdam (*n* = 289)	Utrecht (*n* = 291)	
Agree	49.4 (1232)	**55.9 (133)†**	48.3 (142)	**54.4 (231)†**	45.5 (131)	52.9 (164)	**43.3 (155)†**	**43.3 (125)†**	51.9 (151)	
Neutral	28.8 (719)	23.5 (56)	30.3 (89)	27.5 (117)	33.7 (97)	28.1 (87)	30.7 (110)	31.5 (91)	24.7 (72)	
Disagree	21.7 (542)	20.6 (49)	63 (21.4)	**18.1 (77)†**	20.8 (60)	19 (59)	**26 (93)†**	25.3 (73)	23.4 (68)	
	**Older year medical students can teach younger year medical students well**									
	Total (*n* = 2490)	UvA (*n* = 238)	VU (*n* = 294)	Groningen (*n* = 425)	Leiden (*n* = 288)	Maastricht (*n* = 308)	Nijmegen (*n* = 357)	Rotterdam (*n* = 289)	Utrecht (*n* = 291)	
Agree	63.6 (1584)	68.9 (164)	67.7 (199)	**74.8 (318)†**	**53.1 (153)†**	**55.2 (170)†**	60.8 (217)	**54.7 (158)†**	**70.4 (205)†**	
Neutral	24.3 (604)	22.3 (53)	22.4 (66)	**16.7 (71)†**	26 (75)	**35.1 (108)†**	24.6 (88)	**29.8 (86)†**	**19.6 (57)†**	
Disagree	12.1 (302)	8.8 (21)	9.9 (29)	**8.5 (36)†**	**20.8 (60)†**	9.7 (30)	14.6 (52)	15.6 (45)	10 (29)	
	**I feel well prepared to teach as a physician from the medical school**									
	Total (*n* = 2486)	UvA (*n* = 237)	VU (*n* = 293)	Groningen (*n* = 424)	Leiden (*n* = 288)	Maastricht (*n* = 307)	Nijmegen (*n* = 358)	Rotterdam (*n* = 289)	Utrecht (*n* = 290)	
Agree	26.5 (658)	**19.4 (46)†**	**31.7 (93)†**	**33 (140)†**	**20.8 (60)†**	30.6 (94)	**21.5 (77)†**	**21.5 (62)†**	29.7 (86)	
Neutral	34.5 (858)	32.1 (76)	**41 (120)†**	32.8 (139)	32.3 (93)	35.5 (109)	31.8 (114)	30.1 (87)	41.4 (120)	
Disagree	39 (970)	**48.5 (115)†**	**27.3 (80)†**	**34.2 (145)†**	**46.9 (135)†**	**33.9 (104)†**	**46.6 (167)†**	**48.4 (140)†**	**29 (84)†**	
	**I feel well prepared to teach as a physician based on my own experience**									
	Total (*n* = 2479)	UvA (*n* = 236)	VU (*n* = 292)	Groningen (*n* = 421)	Leiden (*n* = 286)	Maastricht (*n* = 307)	Nijmegen (*n* = 358)	Rotterdam (*n* = 288)	Utrecht (*n* = 291)	
Agree	47.6 (1180)	**39 (92)†**	**55.8 (163)†**	49.9 (210)	49 (140)	48.5 (149)	**41.1 (147)†**	45.8 (132)	50.5 (147)	
Neutral	30.4 (758)	33.1 (78)	27.4 (80)	29.2 (123)	28 (80)	34.9 (107)	27.9 (100)	31.9 (92)	32.3 (94)	
Disagree	22 (545)	**28 (66)†**	**16.8 (49)†**	20.9 (88)	23.1 (66)	**16.6 (51)**	**31 (111)†**	22.2 (64)	**17.2 (50)**	
Missing; n (%)	173–187 (6.5–7)	16–18 (6.3–7.1)	21–23 (6.7–7.4)	29–33 (6.6–7.3)	23–25 (7.4–8)	18–21 (5.5–6.4)	23–24 (6–6.3)	21–22 (6.8–7.1)	22–23 (7–7.3)	

† Significant for *p* < 0.05 (rows)

UvA = Universiteit van Amsterdam

VU = Vrije Universiteit

### Procedure

The survey was part of the annual online survey of the Dutch medical advocacy group (DeGeneeskundestudent) amongst all medical students in the fall of 2017. Participants voluntarily filled out the questionnaire and informed consent was given for anonymous use of the data.

### Data analysis

Before data-analysis we excluded the following participants. Participants with an abbreviated medical study were excluded because they had already finished a wide range of different previous bachelor-studies. Participants who had not filled out the general section were excluded as well. The results were analysed with SPSS version 25. The general section was analysed with descriptive statistics. The study population was compared with available national data on medical students regarding gender, study-phase and university [19, 20]. The questions on the participants view answered on a Likert scale were dichotomised to agree (strongly agree-agree) and disagree (strongly disagree-disagree). In the analysis, we left out the responses to category 3 to get an impression of students’ positive or negative attitude towards peer-teaching and, regarding question 2, to avoid bias from people who have no experience with it giving an opinion. The results were analysed with descriptive statistics. The participants view according to different gender, study-phase or university was analysed with a chi-squared-test or binomial test. The binary question on the participants view was analysed with descriptive statistics. The participants view according to different gender, study-phase or university was analysed with a chi-squared-test. The outcome of all tests was significant if *p* < 0.05.

## Results

### Respondents´ characteristics

The respondents´ characteristics are shown in Table 3. A total of 2666 medical students filled out the survey. The percentage of male respondents was lower than the national average, 23% versus 34%, as well as the percentage of master students, 47% versus 53%. The percentage of respondents from the University of Amsterdam (UvA), Vrije Universiteit (VU) and Rotterdam was slightly lower than the national average, while the percentage of respondents from Groningen, Leiden and Nijmegen was higher than the national average. The distribution of respondents across years of study is similar to the distribution in the overall population.

**Table 3:** Characteristics of participating medical students

	Number of students: % (*n*)*
	Participating medical students
	Total (*n* = 2666)
**Men**	22.7 (606)
**Study year**	
Bachelor 1	22.7 (604)
Bachelor 2	14.8 (395)
Bachelor 3	15.4 (410)
Master 1	17.3 (461)
Master 2	14.8 (394)
Master 3	15.1 (402)
**Master students**	47.1 (1257)
**University**	
Amsterdam (UvA)	9.5 (254)
Amsterdam (VU)	11.8 (315)
Groningen	17 (454)
Leiden	11.7 (311)
Maastricht	12.3 (328)
Nijmegen	14.3 (381)
Rotterdam	11.6 (310)
Utrecht	11.7 (313)

Explanatory notes

*Total population of medical students: 17,329

UvA = Universiteit van Amsterdam

VU = Vrije Universiteit

### View on teaching as a physician and peer-teaching

The results on teaching as a physician and peer-teaching are shown in Tables 1 and 2. Significant results are highlighted in the paragraph below.

### Teaching as a physician

49% of the respondents agrees that teaching is a core responsibility of a physician, while 22% of the respondents disagrees. Male respondents agree more often than female respondents, 58% versus 47%, as well as respondents in the master phase than respondents in the bachelor phase, 64% versus 35%. Agreement of respondents from different universities was between 43% and 56%.

### Peer-teaching

64% of the respondents agrees that senior medical students can educate junior medical students well, while 13% of the respondents disagrees. Respondents in the master phase disagree more often than respondents in the bachelor phase, 13% versus 11%. Agreement of respondents from different universities was between 53% and 75%.

### View on preparation for teaching as a physician

The results on preparation for teaching as a physician by the formal education and respondents’ own experience are shown in Tables 1 and 2. Table 4 shows the respondents own experience with teaching. Significant results are highlighted in the paragraph below.

**Table 4:** Results of the question on own teaching experience

Result*	Number of students; % (n/N)^‡^													
	Do you have teaching experience?													
	Total (*n* = 2476)		Men (*n* = 550)			Woman (*n* = 1926)		Bachelor (*n* = 1257)			Master (*n* = 1219)			
Yes	52.3 (1296)		**58.7 (232)†**			50.5 (973)		41.9 (527)			**63.1 (769)†**			
As student assistent	12.5 (310)		**16 (88)†**			11.5 (222)		6.9 (87)			**18.3 (223)†**			
As part of the curriculum	11.2 (277)		12 (66)			11 (211)		6 (75)			**16.6 (202)†**			
Extracurricular	37.2 (970)		**42 (231)†**			35.8 (689)		32.7 (411)			**509 (41.8)†**			
Missing; n (%)	190 (7.1)		**9.2 (56)†**			6.5 (134)		**10.8 (152)†**			3 (38)			
	**Do you have teaching experience?**													
	Total (*n* = 2476)	UvA (*n* = 236)		VU (*n* = 292)	Groningen (*n* = 419)		Leiden (*n* = 286)		Maastricht (*n* = 307)	Nijmegen (*n* = 358)		Rotterdam (*n* = 287)	Utrecht (*n* = 291)	
Yes	52.3 (1296)	47.5 (112)		54.5 (159)	60.1 (252)		51.7 (148)		56.4 (173)	43.9 (157)		49.5 (142)	52.6 (153)	
As student assistent	12.5 (310)	**6.8 (16)†**		**17.1 (50)†**	**16.2 (68)†**		**16.8 (48)†**		**3.5 (11)†**	10.3 (37)		11.8 (34)	14.8 (46)	
As part of the curriculum	11.2 (277)	8.1 (19)		7.9 (23)	**20.8 (87)†**		8.7 (25)		11.4 (35)	**7 (25)†**		**6.3 (18)†**	**15.5 (45)†**	
Extracurricular	37.2 (970)	36.9 (87)		39.7 (116)	35.3 (148)		35.3 (101)		**45.3 (139)†**	**32.7 (117)†**		39.7 (114)	33.7 (98)	
Missing; n (%)	190 (7.1)	18 (7.1)		23 (7.3)	35 (7.4)		25 (8)		21 (6.4)	23 (6)		23 (7.4)	22 (7)	

† Significant for *p* < 0.05 (rows)

UvA = Universiteit van Amsterdam

VU = Vrije Universiteit

### Formal education

27% of the respondents agrees that the medical education prepares them well for teaching as a physician, while 39% disagrees. Male respondents agree more often than female respondents, 36% versus 24%. Respondents in the master phase disagree more often than respondents in the bachelor phase, 46% versus 33%. Agreement of respondents from different universities was between 19% and 33%.

### Own experience

48% of the respondents agrees that their own experience with teaching prepares them well for teaching as a physician, while 22% disagrees. Male respondents agree more often than female respondents, 62% versus 44%. Respondents in the master phase agree more often than respondents in the bachelor phase, 56% versus 40%. Agreement of respondents from different universities was between 39% and 56%.

52% of the respondents have teaching experience. Male respondents more often have experience than female respondents, 59% versus 51%, as well as respondents in the master phase than the bachelor phase, 63% versus 42%. The percentage of respondents from different universities with teaching experience varies between 44% and 60%.

Of the respondents with teaching experience, 13% have experience as peer-teacher, 11% as part of the formal education and 37% outside the formal education. Male respondents have more experience than female respondents with teaching outside the formal education, 42% versus 36%, and as peer-teacher, 16% versus 12%. Respondents in the master phase have more experience in all manners of teaching than respondents in the bachelor phase. The percentage of respondents from different universities with teaching experience varies, as peer-teacher (4 − 17%), as part of the curriculum (6 − 21%) and outside the formal education (33-45%).

## Discussion

Half of medical students feel that teaching is one of the core tasks of a physician. Unfortunately, only 25% feel well prepared by their medical school for this teaching role. This is in line with the literature that students would benefit from more preparation in this area [21, 22]. It is striking that students who are more advanced in their studies feel less prepared than students who are at the beginning of medical school. The explanation for this may be that older students have more insight into the complexity of the teaching task because they have more experience with the physicians who teach or have had some experience of this themselves. It is contradictory that on the one hand students are aware of their later teaching role and responsibility but on the other hand do not feel adequately prepared for this role. A core task of physicians is to provide knowledge, experiences and skills to different learning groups, e.g. to medical students, patients and other professionals and should therefore be a relevant part of medical education programs.

Almost half of the students feel well prepared for their later teaching role from their own experience. They look for teaching opportunities themselves in anatomy or skills courses or as a secondary job [23]. They agree that their own experience with teaching prepares them well for teaching as a physician. This finding highlights the importance of providing appropriate learning opportunities during medical education. Students engaging as peer-teachers have the chance to gain extracurricular experiences that are relevant not only for professional practice but also to strengthen soft skills and interdisciplinary competencies. Teaching experiences are beneficial in many ways, increase teaching skills, intensify knowledge, increase organizational and communication skills and enhance leading and speaking skills that are relevant for daily practice [6–8, 10, 11, 24].

A large majority of medical students think that older students are good at teaching younger ones. At some universities, students have a more positive image of peer-teaching than at others. It is useful to find out whether these faculties use peer-teaching more as a teaching method.

Thus, medical students’ own views on peer-teaching do not seem to be an impediment to using peer-teaching to learn the role of a teacher. This is also in line with the literature on peer-teaching showing different advantages of learning from other students [11, 25]. First, peer-teachers are closer to the student in experience. Therefore, they can better understand what the students find difficult and they also understand the knowledge level of the students better, compared to, for example, medical specialists [15]. In addition, peer-teachers can create a safe educational climate in which mistakes are allowed and questions can be asked, because peer-teacher are perceived as less threatening [15]. Peer-teachers and students both can profit from peer-teaching settings.

The use of students as teachers can improve teaching capacities and is also connected to economic aspects. To secure high standards in the quality of education in medical schools, peer-teaching programs should be accompanied by training and supervision [11, 17].

A strength of this research project is that it is a cross-section of all Dutch universities and all study years. Therefore, the results give a good picture of the opinion of Dutch medical students. Furthermore, the study focuses on the perspective of medical students. This perspective can be beneficial for gaining insights into medical students’ opinions and for designing adequate learning environments in medical schools. A limitation is that due to the nature of the survey, questionnaires with multiple choice questions, it only provides a global picture. Furthermore, male and bachelor students participated significantly less, which may distort the results. Future research can focus on a comparison between universities with and without formal education in the study program in the area of teaching skills. Furthermore, follow-up research should focus on assessing gender differences. Interviews or focus groups can also provide insight into the motivation and argumentation of the students to gain deeper insights into students’ perceptions. Additionally, further research should also include medical teachers, professionals at medical schools, experts and physicians to gain multiple perspectives. It is also important to focus on the effectiveness of peer-teaching programs in comparison to other learning methods, particularly from a long-term perspective. As teaching skills are a core competence of physicians for daily practice, assessing learning opportunities and methods for physicians in the context of continuing education should also be taken into account.

## Conclusions

Many medical students see teaching as a core task of physicians and are aware of their later teaching role. However, a large proportion of them, especially the students in the last phase of their studies, feel that their medical school program has not adequately prepared them for this role. Instead, there are many students who gain experiences and teaching skills on their own initiative outside medical schools. Preparing medical students for their educational tasks and supporting them in the acquisition of teaching skills should be an essential part of their education. The majority of medical students think that senior students can educate junior medical students well. Therefore, implementing peer-teaching in the curricular of medical schools could be an effective teaching method for learning success. In a broader context, preparing medical students for their teaching role can be beneficial for the patient-medicine relationship and the provision of knowledge and health competency for patients. This study and the literature show that peer teaching, combined with good supervision and feedback, is a good way to prepare medical students for the future teaching role. It is important that medical schools focus on enhancing educational quality and designing beneficial and positive learning environments for best learning outcomes to better prepare medical students for professional life.

## Data Availability

The datasets generated and/or analysed during the current study are not publicly available due to data protection guidelines of the institution but are available from the corresponding author on reasonable request.

## Declarations

### Guidelines

All methods were carried out in accordance with Declaration of Helsinki.

## Abbreviations

SAT: students-as-teachers
UvA: Universiteit van Amsterdam
VU: Vrije Universiteit
